# A canonical FtsZ protein in *Verrucomicrobium spinosum*, a member of the Bacterial phylum *Verrucomicrobia *that also includes tubulin-producing *Prosthecobacter *species

**DOI:** 10.1186/1471-2148-7-37

**Published:** 2007-03-12

**Authors:** Benjamin Yee, Feras F Lafi, Brian Oakley, James T Staley, John A Fuerst

**Affiliations:** 1 School of Molecular and Microbial Sciences, University of Queensland, Brisbane, Queensland 4072, Australia; 2 Department of Microbiology, University of Washington, Seattle, WA 98195, USA

## Abstract

**Background:**

The origin and evolution of the homologous GTP-binding cytoskeletal proteins FtsZ typical of Bacteria and tubulin characteristic of eukaryotes is a major question in molecular evolutionary biology. Both FtsZ and tubulin are central to key cell biology processes – bacterial septation and cell division in the case of FtsZ and in the case of tubulins the function of microtubules necessary for mitosis and other key cytoskeleton-dependent processes in eukaryotes. The origin of tubulin in particular is of significance to models for eukaryote origins. Most members of domain Bacteria possess FtsZ, but bacteria in genus *Prosthecobacter *of the phylum *Verrucomicrobia *form a key exception, possessing tubulin homologs BtubA and BtubB. It is therefore of interest to know whether other members of phylum *Verrucomicrobia *possess FtsZ or tubulin as their FtsZ-tubulin gene family representative.

**Results:**

*Verrucomicrobium spinosum*, a member of Phylum *Verrucomicrobia *of domain Bacteria, has been found to possess a gene for a protein homologous to the cytoskeletal protein FtsZ. The deduced amino acid sequence has sequence signatures and predicted secondary structure characteristic for FtsZ rather than tubulin, but phylogenetic trees and sequence analysis indicate that it is divergent from all other known FtsZ sequences in members of domain Bacteria. The FtsZ gene of *V. spinosum *is located within a *dcw *gene cluster exhibiting gene order conservation known to contribute to the divisome in other Bacteria and comparable to these clusters in other Bacteria, suggesting a similar functional role.

**Conclusion:**

*Verrucomicrobium spinosum *has been found to possess a gene for a protein homologous to the cytoskeletal protein FtsZ. The results suggest the functional as well as structural homology of the *V. spinosum *FtsZ to the FtsZs of other Bacteria implying its involvement in cell septum formation during division. Thus, both bacteria-like FtsZ and eukaryote-like tubulin cytoskeletal homologs occur in different species of the phylum *Verrucomicrobia *of domain Bacteria, a result with potential major implications for understanding evolution of tubulin-like cytoskeletal proteins and the origin of eukaryote tubulins.

## Background

Both eukaryotes and prokaryotes are now known to possess cytoskeletal proteins, including tubulin components of the eukaryotic microtubules important in mitosis and the tubulin homolog FtsZ of bacteria which forms a septal Z-ring during bacterial cell division[1,2]. Most bacteria possess a cell division protein more closely related to FtsZ, the homolog of tubulin present almost universally within bacteria, than to the tubulin characteristic of eukaryotes [3-5]. Some rare bacterial species appear to lack any tubulin or FtsZ homolog at all [4]. Both FtsZ and tubulin are significant for evolutionary cell biology since FtsZ is pivotal to cell division in most bacteria and tubulin is a cytoskeletal eukaryote signature protein [6]central to models for origins of eukaryote cell organization [7,8]. Tubulin has been confirmed to be the homolog of FtsZ at the 3D structural level by crystallography [9,10]. Both proteins can polymerize into filaments, in the case of tubulin as a heterodimer of alpha and beta subunits that forms the basis *in vivo *of eukaryote cytoskeletal microtubules central to many processes of eukaryote cell biology, and in the case of FtsZ into protofilaments *in vitro *and septal rings *in vivo *[2].

The genus *Prosthecobacter *is a member of the phylogenetically divergent phylum *Verrucomicrobia *[11] which forms a superphylum with other phyla *Planctomycetes *and *Chlamydia *of the domain Bacteria [12], and species of this genus such as *P. dejongeii*, possess the proteins BtubA and BtubB more closely related to eukaryotic tubulin than to bacterial FtsZ [13]. BtubA and BtubB appear to be genuine tubulins, and can form three-dimensional bundles of up to 30 protofilaments in the presence of GTP *in vitro *[14]. *Prosthecobacter dejongeii*, however, does not apparently possess FtsZ [14]. It would be expected that other members of the phylum *Verrucomicrobia *would also possess such a tubulin homolog e.g. if a last common ancestor of the verrucomicrobia also possessed such a homolog. We therefore searched the draft genome of *Verrucomicrobium spinosum *for the presence of genes similar to cytoskeletal proteins such as FtsZ and tubulin, and revealed the presence of a gene coding for a protein with primary and secondary structural characteristics and evolutionary relationships consistent with closer relationship to genes for bacterial FtsZ than to those for eukaryotic tubulins.

## Results and discussion

A putative gene (Genbank: DQ845344) which appears to code for an ortholog of bacterial FtsZ, a cytoskeletal protein involved in cell division, was amplified to confirm the sequence from examining the draft genome of *Verrucomicrobium spinosum*. The translated protein VerFtsZ from this putative gene is 673 amino acids long, and application of FingerPRINTScan showed that VerFtsZ contains each of the six motifs specific to bacterial FtsZ [15]. Consistent with this, application of GeneFIND for gene family identification showed a highly probable relationship to the FtsZ and FtsZ1 superfamily[16].

**Figure 1:**
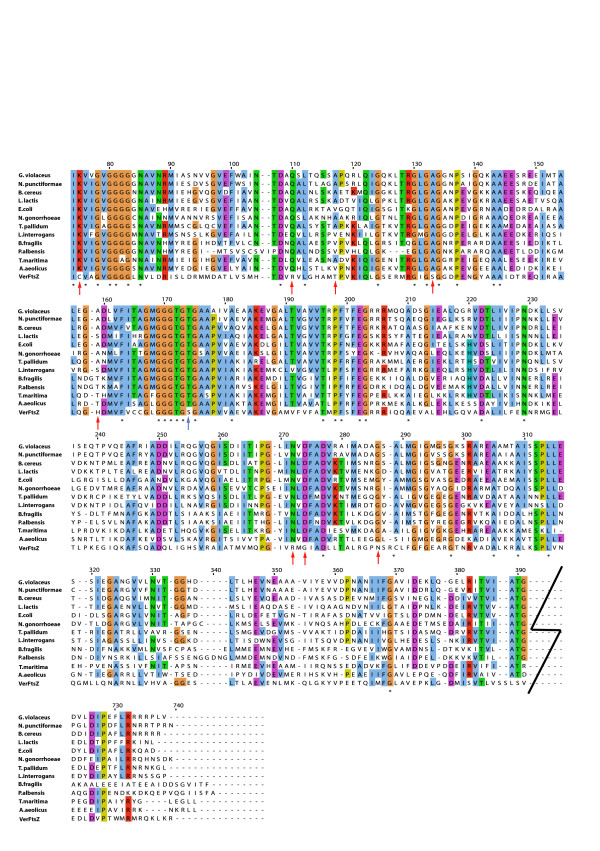
Sequence alignment of VerFtsZ of *Verrucomicrobium spinosum *with reference amino acid sequences of FtsZ from representative Bacteria with highlight of residues based on an identity threshold of at least 50% across taxa. The first and last block of the alignment shows the N-terminal and C-terminal region respectively while the middle four blocks form the core region (the first 70 poorly alignable residues of the N-terminal region are not shown). Identities are denoted with a '*' below the amino acid position. Red arrows indicate amino acid positions at which the sequence of *Verrucomicrobium spinosum *differs from the FtsZ sequences of all other Bacterial phyla in the alignment. The blue arrow indicates the position of uncommon Serine within the tubulin signature motif. L.lactis, *Lactococcus lactis*; N.gonorrhoeae, *Neisseria gonorrhoeae*; B. fragilis, *Bacteroides fragilis*; P. albensis, *Prevotella albensis*; N. punctiformae, *Nostoc punctiformae*; G. violaceus, *Gloeobacter violaceus*; B. cereus, *Bacillus cereus*; E. coli, *Escherichia coli*; T. pallidum, *Treponema pallidum*; L. interrogans, *Leptospira interrogans*; T. maritima, *Thermotoga maritima*; A. aeolicus, *Aquifex aeolicus; *VerFtsZ, *Verrucomicrobium spinosum*.

The core region of FtsZ in bacteria starts with a highly conserved isoleucine with only 14 changes at the position in 225 sequences from different organisms and ends with a recognizable version of the amino acid sequence LVITG. The core region of bacterial FtsZ also contains a sequence similar to the highly conserved tubulin signature motif (PROSITE motif PS00227: ([S/A/G]GGTG [S/A/T]G). VerFtsZ aligns perfectly with the conserved isoleucine of other FtsZ sequences at the start of the core region and also contains the tubulin signature – as manifested by the sequence GGGTGSG (Fig. 1). However, VerFtsZ shows a fairly divergent sequence at the end of the core region (Fig. 1), which was observed in seven sequences of Archaea FtsZ3, as well as in *Mycoplasma pneumoniae *and *Mycoplasma genitalium *FtsZ. The C-terminal region of bacteria, which interacts directly with FtsA, lacks a classical consensus sequence due to substitutions but a sequence logo has been derived from an alignment to display the pattern of protein sequences at the C-terminal [4], and there is some relative conservation of amino acids at particular positions. The C-terminal of VerFtsZ seemed to vary slightly from this sequence logo but it does share the highly conserved Proline position (position number 728 in Fig. 1) with other FtsZ sequences.

A more detailed examination of the core region of VerFtsZ reveals significant divergence at amino acid positions otherwise conserved among other phyla. There are 8 instances (red arrows in Fig. 1) of amino acid changes to another amino acid of different physico-chemical properties from those conserved completely in the other sequences, which could imply possible structural and physiochemical significance e.g. hydrophilic to hydrophobic for the change of lysine to cysteine at position 75. The amino acid serine within the tubulin signature motif (highlighted with blue arrow at position 174) presents yet another significant difference. An examination of available database for the tubulin signature motif at PROSITE, and that compiled by Vaughan *et al., *revealed that a serine at this position is present only in the FtsZ of the alpha-proteobacterium *Magnetospirillium magnetotacticum*, plastids of some members under Viridiplantae and the Archaea *Halobacterium *species *NRC-1*, but highly conserved throughout the eukaryotic tubulins and also in BtubA and BtubB of *Prosthecobacter*.

Preliminary deduction of VerFtsZ protein structure from secondary structure comparisons using Cn3D database at NCBI resulted in reasonable alignment to the structural model of 1FSZ for FtsZ of *Methanococcus jannaschii *from Protein Data Bank (note that this species is synonymous with *Methanocaldococcus jannaschii*, the name used in some other databases employed for this study).

**Figure 2:**
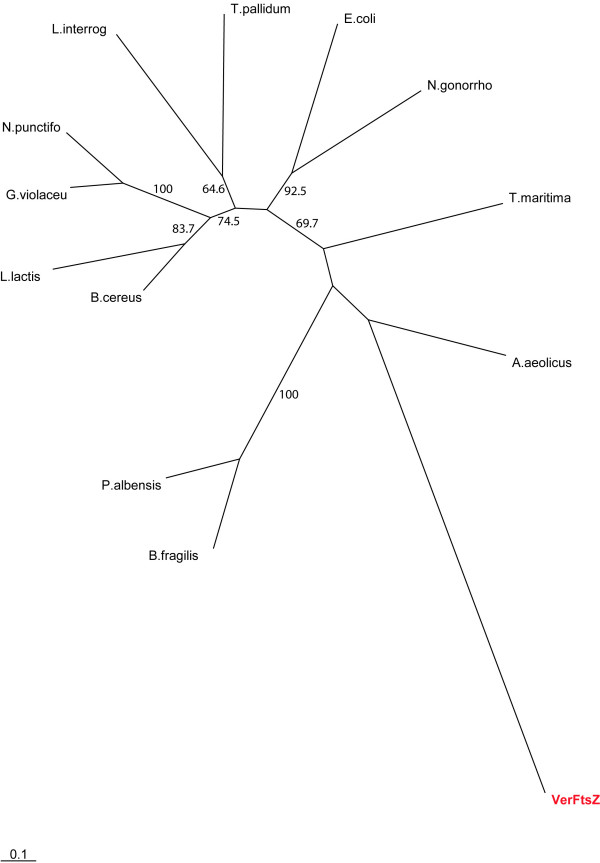
Maximum Likelihood phylogenetic tree including VerFtsZ (red text) and reference FtsZ sequences from Bacteria. The tree was derived via TreeFinder and bootstrap confidence values are based on 1,000 replicates. Scale bar represents 0.1 substitutions per site. L.lactis, *Lactococcus lactis*; N.gonorrho, *Neisseria gonorrhoeae*; B. fragilis, *Bacteroides fragilis*; P. albensis, *Prevotella albensis*; N. punctifo, *Nostoc punctiformae*; G. violaceu, *Gloeobacter violaceus*; B. cereus, *Bacillus cereus*; E. coli, *Escherichia coli*; T. pallidum, *Treponema pallidum*; L. interrog, *Leptospira interrogans*; T. maritima, *Thermotoga maritima*; A. aeolicus, *Aquifex aeolicus; *VerFtsZ, *Verrucomicrobium spinosum* FtsZ.

In order to investigate the relationship of VerFtsZ to FtsZ of other bacteria, we performed phylogenetic analysis using the alignment in Fig 1 which consists of a set of reference sequences of FtsZ of bacteria of different phyla. The alignment contains the functional core and C-terminal domains of FtsZ while the variable spacer region has been omitted. Phylogenetic analysis of an alignment including VerFtsZ and reference sequences of FtsZ from bacteria was performed using Treefinder [17]. In the analysis (Fig. 2), the long branch of VerFtsZ confirms its divergence from the other representatives of FtsZ among the domain Bacteria with no apparent implication of horizontal gene transfer from any existing bacterial phyla.

VerFtsZ displays lower similarity to FtsZs of other Bacterial phyla than the similarity of the FtsZs of any of those phylum representatives to the FtsZs from any other Bacterial phylum. For example, when calculated using an alignment without the variable spacer region, the FtsZ of *Aquifex aeolicus*, widely considered a deep-branching bacterium, exhibits a similarity to VerFtsZ of only 36.8%, yet shows a similarity of at least 42% to the other representative FtsZs, and other Bacterial FtsZs often display greater similarity to any other Bacterial FtsZs than to VerFtsZ.

**Figure 3:**
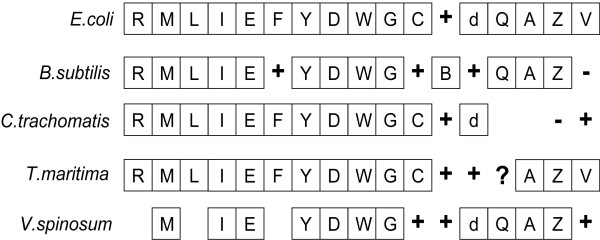
Order of *dcw *cluster proteins in *V.spinosum *as compared to those of selected reference bacteria [39]. The key to one letter gene symbols are as follows : R, mraZ; M, *mraW*; L, ftsL; I, *ftsI*; E, *murE*; F, *murF*; Y, *mraY*; D, *murD*; W, *ftsW*; G, *murG*; C, *murC*; B, *murB; *d, *ddlB*; Q, *ftsQ*; A, *ftsA*; Z, *ftsZ*; V, *envA*(*lpxC*). Full species names are *E.coli, Escherichia coli*; *B.subtilis, Bacillus subtilis*; *C.trachomatis, Chlamydia trachomatis*; *T.maritima, Thermotoga maritima*; *V.spinosum, Verrucomicrobium spinosum*. '+' symbols represent a homologue is present at a different location within the genome, '-' symbols represent no recognizable homologue found and is only used where complete genome sequences are available, '?' symbols indicate a possible homologue. It should be noted that although genes are often shown as if contiguous, in some cases genes are separated from the next downstream e.g. in *C. trachomatis, murE *is separated from *murF*. Also, some genes are actually contiguous but a + sign intervenes only to indicate that a homolog missing in an expected position in between them is present but in another part of the genome.

In other Bacteria, the *ftsZ *gene is known to form part of the *dcw *gene cluster which possesses other genes coding for proteins concerned with cell division coordinated with FtsZ in the divisome, such as FtsA, FtsI, FtsW, FtsQ and several genes concerned with cell wall synthesis such as the peptidoglycan synthesis *mur *genes *murG*, *murD *and *murE *[18]. *V.spinosum *contains an almost complete set of proteins of the *dcw *gene cluster (Fig. 3), with only MraZ and FtsL missing from the cluster. The genes for MurB, MurC and EnvA are absent from the *V. spinosum dcw *cluster but corresponding sequences are present elsewhere in the genome of this species. The order of the genes in the *dcw *cluster in *V. spinosum *can be seen to be similar to the gene order of the *dcw *clusters of bacteria in several distinct phyla (as represented by *Escherichia coli, Bacillus subtilis, Thermotoga maritima *and *Chlamydia trachomatis *in Fig. 3, members of the phyla *Proteobacteria*, *Firmicutes*, *Thermotoga*, and *Chlamydia *respectively). Despite sharing membership of a potential superphylum with *Chlamydia trachomatis*, the *dcw *cluster of *V. spinosum*, like *E. coli *and *B. subtilis*, possesses FtsQ, FtsA and FtsZ in that same order, while these are missing from the *dcw *cluster of *C. trachomatis*, an organism known to possess no FtsZ.

The organization of the *ftsZ *gene of *V. spinosum *within a gene cluster known to be concerned with formation of a septation divisome and related cell wall synthesis in *E. coli *suggests that the verrucomicrobial FtsZ protein also participates in a multi-protein divisome organizing septum synthesis.

## Conclusion

It is clear from the above analysis that VerFtsZ of the verrucomicrobia member *Verrucomicrobium spinosum *is a true ortholog of other FtsZ sequences from Bacteria, and differs markedly from the tubulin homologs BtubA and BtubB of *Prosthecobacter dejongeii*. BtubA and BtubB have been shown to form a similar heterodimer to αβ-tubulin and seem to be bacterial tubulins fundamentally distinct from FtsZ while sharing the features common to the FtsZ-tubulin family [19]. This difference between the *V. spinosum *FtsZ and the *P. dejongeii *tubulins is of considerable evolutionary interest, since clearly from our analysis, these two markedly different proteins occur in two species within the same phylum *Verrucomicrobia *of domain Bacteria. One would expect from phylogenetic considerations that members of the same monophyletic phylum level group within the Bacteria would possess orthologs of only FtsZ or tubulin but that both of these closely related but quite distinct orthologs would not be present in the same phylum. The occurrence of both FtsZ and tubulin homologs within different genera of the same phylum can be explained either by a gene loss and replacement by a lateral gene transfer event or by a close relationship of the FtsZ and tubulins found in this phylum to a last common ancestor possessing a protein with similarities to both. It has been pointed out from structural considerations that horizontal gene transfer is one explanation for the origin of the tubulin homologs BtubB and BtubA in *P. dejongeii *[19].

The unexpected occurrence of both FtsZ and tubulin cytoskeletal proteins in one bacterial phylum could be explained in several ways. Both proteins could have been present in the common ancestor of *Verrucomicrobium* and *Prosthecobacter* and then may have been differentially lost or have significantly diverged. Alternatively, horizontal transfer from proto-eukaryotes or eukaryotes to Prosthecobacter may account for tubulin in this genus and this is supported by genome data suggesting a largely bacterial nature for *P.dejongeii *genes [19].

*Verrucomicrobium spinosum *and *Prosthecobacter dejongeii *form a relatively shallow clade within the verrucomicrobia according to other genes. The 16S rRNA similarity between *Verrucomicrobium spinosum *and *Prosthecobacter dejongeii *as well as all other species of *Prosthecobacter *is quite high at 91%, relative to the similarity of members of both these genera to deeper-branching species of verrucomicrobia such as *Victivallis vadensis *(76% in each case) or *Opitutus terrae *(80%). This should be more compatible with a late lateral gene transfer of tubulin genes into *Prosthecobacter *species than an ancient gene duplication in a verrucomicrobial ancestor (since retention of tubulin in both members of a resulting lineage or clade within verrucomicrobia would be expected if inherited vertically from a common ancestor).

Lateral transfer of tubulin genes seems more likely than transfer of FtsZ genes since a whole *dcw *cluster would have to be transferred. Loss and replacement of a complete multi-gene cell division mechanism necessary for viability of a cell population seems an unlikely scenario in case of one incorporating either FtsZ or tubulin, but the similarity of ancestral FtZs and tubulins could have meant such ancestral proteins could have been easily interchangeable within a common gene organization. Otherwise a version of the 'complexity hypothesis' [20]relating to cell division as a complex system of interacting proteins suggests that it would be difficult to replace FtsZ within a *dcw *cluster with a more evolved tubulin. From existing draft genome sequence, it appears that the significant gene for the FtsA protein important for interaction with FtsZ is missing from *P. dejongeii*, though genes of the *dcw *cluster concerned with peptidoglycan synthesis such as *murD *are present. *P. dejongeii *also seems to have a gene for FtsW, known to interact with peptidoglycan synthesis proteins[21]. Significantly, the tubulin genes in this species are organized in an operon along with a kinesin-like gene, suggesting they are not a part of the *dcw *cluster [13]. Co-expression of *btuba *and *btubb *in *E. coli *results in either prominent rods running along the cell length or loose spirals so this suggest that these proteins by themselves may be able to form cytoskeletal filaments in vivo [14].

Tubulin genes may be found in other verrucomicrobia branching more deeply than *Prosthecobacter *species within the verrucomicrobia. This is suggested by the report via anti-tubulin antibodies of tubulin in the 'epixenosomes', verrucomicrobial symbionts of marine ciliate protozoan *Euplotidium *[22]. Epixenosomes are members of subdivision 4 of the phylum *Verrucomicrobia *[23,24] along with the cultured *Opitutus terrae*. The distribution of genes for both tubulins and FtsZ must be determined in a wider selection of verrucomicrobia among all 5 subdivisions distinguished[25,26], e.g. cultured members of genera *Opitutus *and *Chthoniobacter*, in order to determine an optimal basis for further investigations of their evolution via data from genomics and cell biology. Since genome data for *Verrucomicrobium spinosum *and *Prosthecobacter dejongeii *is only in draft form so far, there is a possibility that either or both of these species possess both FtsZ and tubulin, and this should be confirmed or refuted by annotation of completed genomes for these species.

Discovery of a verrucomicrobial FtsZ in at least one genus and species combined with our existing knowledge of a tubulin in another verrucomicrobial genus reveals that two markedly different members of the tubulin-FtsZ cytoskeletal protein family occur within the same clade of the same bacterial phylum. This result may be of potential significance for our understanding of eukaryote and Bacterial cytoskeleton evolution, since such occurrence may suggest new models for cytoskeletal protein evolution e.g. derivation of both FtsZ and tubulin from an ancestral protein or proteins present in a Bacterial ancestor, or lateral transfer of cytoskeletal genes between domains at early stages in their evolution. Members of the phylum *Verrucomicrobia *may hold a key to the ancient molecular evolutionary stages by which tubulins and FtsZ's diverged and differentiated, or alternatively, the trace of an early event in the gene transfer network between prokaryotes and eukaryotes which may have preserved an early 'snapshot' of the evolution of eukaryote cytoskeletal proteins. Such models may be testable via a combination of further genomic and bioinformatic analysis of existing and newly discovered verrucomicrobia.

## Methods

### Bacterial strains and culture

Cultures of *Verrucomicrobium spinosum *DSM 4136 were grown aerobically at room temperature in modified medium B agar [27].

### DNA extraction

DNA was extracted from *Verrucomicrobium spinosum cultures *by using the Instagene matrix (Bio-Rad).

### Amplification of *verftsz*

The *verftsz *gene was amplified from single *V. spinosum *colonies via PCR. PCR primers used for the PCR (5' – 3') were as follows: AGTATTCCAGTGTAACCCG (forward) and GTCTCTTGAAGTGAAGGCTC (reverse). PCR was carried out with an initial denaturation at 96°C for 3 mins, followed by 32 cycles of 94°C for 1 min 30 sec, annealing at 58°C for 1 min, and extension at 72°C for 1 min 30 sec followed by a final extension step at 72°C for 10 mins. Primers used for sequencing were the same as for PCR with the addition of two internal sequencing primers (5' – 3') CTCCTTTGAGGGGCGTCGT (forward) and TGTCCCCTTCATCTTCCTCA (reverse).

### Sequence analysis

The putative protein hereby termed VerFtsZ was analysed for signature motifs using the program FingerPRINTScan [28,29]. Gene family identification was done through Gene Family Identification Network Design (GeneFIND) [30]. Secondary structure of putative sequences was determined using the three-dimensional molecular structure viewer program Cn3D 4.1 [31] by structural homology matching against 1FSZ, the FtsZ structure of *Methanococcus jannaschii *[9]in the Protein Data Bank at NCBI.

### Conserved region analysis

Regions of conservation such as the core and C-terminal regions were analysed based on a published analysis of FtsZ [4], and an alignment of VerFtsZ against published reference sequences of Bacterial amino acid sequences was performed using CLUSTALX. V. 1.8 [32]

### Phylogenetic analysis

Alignment of VerFtsZ against reference sequences of Bacterial FtsZs was performed using CLUSTALX. V. 1.8 [32]. Optimization of the alignment i.e removal of variable spacer region and alignment of C-terminal region was performed using GeneDoc [33] The Modelgenerator [34] was used to obtain the model and parameters for the likelihood analysis for the alignment of VerFtsZ and FtsZ. Modelgenerator selected WAG + G as the best model for the maximum likelihood analyses based on the Akaike Information Criterion 1, Akaike Information Criterion 2, and Bayesian Information Criterion. A maximum likelihood tree was generated using TreeFinder [17]. Bootstrap analysis using 1,000 replicates with 4 gamma categories under the WAG substitution matrix [35] under the assumption of gamma-distributed, substitution rate variation [36] was performed on the tree with the highest likelihood score. Similarity values were calculated within PHYLIP Protdist advanced options within Institut Pasteur's Software for Biology site [37].

### In silico analysis

#### Homology search

Gene searches utilized the *Verrucomicrobium spinosum *genome made available for public release by TIGR (The Institute for Genomic Research, Rockville, Maryland). Searches for the proteins of the *dcw *gene cluster (mraW, mraY, mraZ, murB, murC, murD, murE, murF, murG, FtsA, FtsI, FtsL, FtsQ, FtsW, FtsZ and ddlB) were conducted using query amino acid sequences from *Escherichia coli *and the similarity search function (tblastn) at The Institute of Genome Research (TIGR).

## Authors' contributions

BY carried out the bioinformatic and phylogenetic analyses and designed PCR primers, and FFL participated in the phylogenetic analysis. BO carried out gene retrieval and sequencing from *V. spinosum*, JAF conceived of the study, participated in its design and coordination, and drafted the manuscript with BY, and JTS supplied *V. spinosum *cultures and sequence data on *P. dejongeii*. All authors read and approved the manuscript.

## References

[B1] Margolin W (2000). Themes and variations in prokaryotic cell division. FEMS Microbiol Rev.

[B2] Margolin W (2005). FtsZ and the division of prokaryotic cells and organelles. Nat Rev Mol Cell Biol.

[B3] Addinall SG, Holland B (2002). The tubulin ancestor, FtsZ, draughtsman, designer and driving force for bacterial cytokinesis. J Mol Biol.

[B4] Vaughan S, Wickstead B, Gull K, Addinall SG (2004). Molecular evolution of FtsZ protein sequences encoded within the genomes of archaea, bacteria, and eukaryota. J Mol Evol.

[B5] Lowe J, van den Ent F, Amos LA (2004). Molecules of the bacterial cytoskeleton. Annu Rev Biophys Biomol Struct.

[B6] Hartman H, Fedorov A (2002). The origin of the eukaryotic cell: a genomic investigation. Proc Natl Acad Sci U S A.

[B7] Faguy DM, Doolittle WF (1998). Cytoskeletal proteins: The evolution of cell division. Curr Biol.

[B8] Cavalier-Smith T (2006). Cell evolution and Earth history: stasis and revolution. Philos Trans R Soc Lond B Biol Sci.

[B9] Lowe J, Amos LA (1998). Crystal structure of the bacterial cell-division protein FtsZ. Nature.

[B10] Nogales E, Wolf SG, Downing KH (1998). Structure of the alpha beta tubulin dimer by electron crystallography. Nature.

[B11] Hedlund BP, Gosink JJ, Staley JT (1997). *Verrucomicrobia* div. nov., a new division of the bacteria containing three new species of *Prosthecobacter*. Anton Leeuw Int J G.

[B12] Wagner M, Horn M (2006). The Planctomycetes, Verrucomicrobia, Chlamydiae and sister phyla comprise a superphylum with biotechnological and medical relevance. Curr Opin Biotechnol.

[B13] Jenkins C, Samudrala R, Anderson I, Hedlund BP, Petroni G, Michailova N, Pinel N, Overbeek R, Rosati G, Staley JT (2002). Genes for the cytoskeletal protein tubulin in the bacterial genus Prosthecobacter. Proc Natl Acad Sci U S A.

[B14] Sontag CA, Staley JT, Erickson HP (2005). In vitro assembly and GTP hydrolysis by bacterial tubulins BtubA and BtubB. Journal of Cell Biology.

[B15] Attwood TK (2002). The PRINTS database: a resource for identification of protein families. Brief Bioinform.

[B16] Barker WC, Pfeiffer F, George DG (1996). Superfamily classification in PIR-International Protein Sequence Database. Methods Enzymol.

[B17] Jobb G, von Haeseler A, Strimmer K (2004). TREEFINDER: a powerful graphical analysis environment for molecular phylogenetics. BMC Evol Biol.

[B18] Vicente M, Gomez MJ, Ayala JA (1998). Regulation of transcription of cell division genes in the *Escherichia coli* dcw cluster. Cell Mol Life Sci.

[B19] Schlieper D, Oliva MA, Andreu JM, Lowe J (2005). Structure of bacterial tubulin BtubA/B: Evidence for horizontal gene transfer. Proceedings of the National Academy of Sciences of the United States of America.

[B20] Jain R, Rivera MC, Lake JA (1999). Horizontal gene transfer among genomes: the complexity hypothesis. Proc Natl Acad Sci U S A.

[B21] Pastoret S, Fraipont C, den Blaauwen T, Wolf B, Aarsman ME, Piette A, Thomas A, Brasseur R, Nguyen-Disteche M (2004). Functional analysis of the cell division protein FtsW of *Escherichia coli*. J Bacteriol.

[B22] Rosati G, Lenzi P, Franco V (1993). Epixenosomes peculiar epibionts of the protozoan ciliate *Euplotidium itoi* - do their cytoplasmic tubules consist of tubulin. Micron.

[B23] Petroni G, Spring S, Schleifer KH, Verni F, Rosati G (2000). Defensive extrusive ectosymbionts of *Euplotidium* (Ciliophora) that contain microtubule-like structures are bacteria related to *Verrucomicrobia*. Proc Natl Acad Sci U S A.

[B24] Chin KJ, Liesack W, Janssen PH (2001). *Opitutus terrae* gen. nov., sp. nov., to accommodate novel strains of the division 'Verrucomicrobia' isolated from rice paddy soil. Int J Syst Evol Microbiol.

[B25] Sangwan P, Chen X, Hugenholtz P, Janssen PH (2004). *Chthoniobacter flavus* gen. nov., sp. nov., the first pure-culture representative of subdivision two, *Spartobacteria* classis nov., of the phylum *Verrucomicrobia*. Appl Environ Microbiol.

[B26] Hugenholtz P, Goebel BM, Pace NR (1998). Impact of culture-independent studies on the emerging phylogenetic view of bacterial diversity. J Bacteriol.

[B27] Staley JT, Mandel M (1973). Deoxyribonucleic acid base composition of *Prosthecomicrobium* and *Ancalomicrobium* strains. International Journal of Systematic Bacteriology.

[B28] P-val FingerPRINTScan. http://www.bioinf.man.ac.uk/ fingerPRINTScan/.

[B29] Scordis P, Flower DR, Attwood TK (1999). FingerPRINTScan: intelligent searching of the PRINTS motif database. Bioinformatics.

[B30] Wu CH, Shivakumar S, Shivakumar CV, Chen SC (1998). GeneFIND web server for protein family identification and information retrieval. Bioinformatics.

[B31] Hogue CWV (1997). Cn3D: a new generation of three-dimensional molecular structure viewer. Trends Biochem Sci.

[B32] Thompson JD, Gibson TJ, Plewniak F, Jeanmougin F, Higgins DG (1997). The CLUSTAL_X windows interface: flexible strategies for multiple sequence alignment aided by quality analysis tools. Nucleic Acids Res.

[B33] Nicholas KB, Jr. NHB, Deerfield DWII (1997). GeneDoc: Analysis and Visualization of Genetic Variation,. EMBNEWNEWS.

[B34] Keane TM, Creevey CJ, Pentony MM, Naughton TJ, McLnerney JO (2006). Assessment of methods for amino acid matrix selection and their use on empirical data shows that ad hoc assumptions for choice of matrix are not justified. BMC Evol Biol.

[B35] Whelan S, Goldman N (2001). A general empirical model of protein evolution derived from multiple protein families using a maximum-likelihood approach. Mol Biol Evol.

[B36] Yang ZH (1993). Maximum-likelihood estimation of phylogeny from DNA sequences when substitution rates differ over sites. Mol Biol Evol.

[B37] Institut Pasteur. http://www.pasteur.fr/english.html.

[B38] The Institute for Genomic Research (TIGR). http://www.tigr.org.

[B39] Nikolaichik YA, Donachie WD (2000). Conservation of gene order amongst cell wall and cell division genes in Eubacteria, and ribosomal genes in Eubacteria and Eukaryotic organelles. Genetica.

